# Brain-derived neurotrophic factor gene polymorphism rs6265 and elite athlete status in four independent populations

**DOI:** 10.5114/biolsport.2025.146786

**Published:** 2025-01-20

**Authors:** Gabija Anikevičiūtė, Alina Urnikytė, Myosotis Massidda, Carla Maria Calò, Filippo Tocco, Mizuki Takaragawa, Eri Miyamoto-Mikami, Haruka Murakami, Motohiko Miyachi, Noriyuki Fuku, Kinga Humińska-Lisowska, Kinga Łosińska, Pawel Cięszczyk, Valentina Ginevičienė

**Affiliations:** 1 Translational health research Institute, Faculty of Medicine, Vilnius University, Lithuania; 2 Department of Medical Sciences and Public Health, University of Cagliari, Italy; 3 Department of Life and Environmental Sciences, University of Cagliari, Italy; 4 Graduate School of Health and Sports Science, Juntendo University, Japan; 5 College of Sport and Health Science, Ritsumeikan University, Japan; 6 Faculty of Sport Sciences, Waseda University, Japan; 7 Department of Physical Education, Gdansk University of Physical Education and Sport, Poland

**Keywords:** elite athlete, case-control study, polymorphism, BDNF, rs6265

## Abstract

This research aimed to investigate the association of the BDNF rs6265 polymorphism with elite athletic status in four different populations: Japanese and European Caucasian cohorts from Italy, Poland, and Lithuania. A total of 1,644 professional athletes (868 Japanese, 177 Italian, 369 Polish, 230 Lithuanian) and 1,948 non-athlete controls (healthy, unrelated 821 Japanese, 102 Italian, 371 Polish, and 654 Lithuanian individuals) were genotyped. The athletes were stratified into endurance-oriented, sprint/power-oriented, and team sports groups. Statistical analysis was performed using R version 4.2.0. The results showed that the allele/genotype distribution of BDNF rs6265 was significantly different between the athlete and control groups and varied across all analysed populations. The minor A allele was significantly more common in the Japanese compared to Europeans, and the G allele/GG genotype was significantly more prevalent in Polish and Lithuanian individuals compared to Italians. European athletes were less likely to have the rare AA genotype than sedentary controls. The GG genotype was more prevalent among Italian athletes, particularly those in team sports, who were about twice as likely to have the GG genotype compared to controls. Lithuanian athletes were more likely to have the GA genotype compared to controls. This was especially true for Lithuanian sprint/power athletes, who had a twofold greater probability of having the GA genotype compared to controls. The BDNF rs6265 variant indicates genetic differences across the four populations (ancestry-relevant heterogeneity) and highlights its potential influence on elite athletic status for the Italian (GG genotype) and Lithuanian (GA genotype) populations.

## INTRODUCTION

The importance of genetics in contemporary sports increases with every year. The main achievement in sports genetics is the determination of genetic markers that are significant for sports training, medicine, and rehabilitation [1]. Currently, there is evidence that one of the potential gene candidates is the *BDNF* gene (and its variant), which encodes brain-derived neurotrophic factor (BDNF). *BDNF* (position chr11: 27654893-27722058, GRCh38.p14) expression and function are highly tissue-dependent. While *BDNF* is well known for its importance in the brain and nervous system, where it supports neuronal growth, differentiation, and maintenance [2], it is also expressed in other tissues, such as the heart, muscle, and even adipose tissue [3]. Research with animals and humans has demonstrated the multifactorial role of BDNF in various cognitive functions (memory, learning), emotional control, personality traits and eating behaviour [4]. *BDNF* gene expression is often activity-dependent, meaning it can be upregulated or downregulated in response to neural activity [5]. Furthermore, *BDNF* has been linked to exercise and sports performance due to its effects on neuroplasticity and brain function [6, 7]. Regular physical activity increases BDNF levels in the brain, improving cognitive function, mood, and overall mental well-being, potentially benefiting athletes in optimization of physical performance [8, 9]. Intense aerobic and anaerobic sports activities lead to an increase in *BDNF* and other myokines (produced and expressed by myofibers during exercise) levels in the blood serum, which reflects the body’s adaptive response to exercise stress and contributes to the maintenance of general physical and psychological health [10–12]. Myokines (including BDNF) mediate communication between muscles and other organs (such as the brain, liver, adipose tissue, and vascular system) and within the muscle itself [13]. These molecules are important for normal muscle function, including movement, support, and other biomechanical processes. Their interaction ensures that the muscles perform their functions efficiently, responding to nerve impulses and regulating the processes of contraction and relaxation [13].

The *BDNF* gene has many single-nucleotide polymorphisms (SNPs), and one of them in particular, rs6265 (NM_001709.5 (*BDNF*): c.196G > A, p.Val66Met), attracts the attention of researchers due to its potential implications for a wide range of physiological and psychological processes, including those related to exercise and sports performance [3]. This *BDNF* common (G/A) nonsynonymous polymorphism, which influences the substitution of valine (Val) for methionine (Met) at codon 66 in the amino acid sequence, affects gene expression by resulting in the dysregulation of microRNA, which in turn affects downstream mRNA levels, resulting in altered BDNF protein expression. Thus, the *BDNF* rs6265 polymorphism has significant effects on protein structure, function, and intracellular processes, influencing self-assembly processes, protein stability, and intracellular sorting [14]. It impacts the levels of BDNF circulating in the bloodstream and at the same time the neuronal adaptation and response, as well as the ability to influence fat metabolism, insulin sensitivity, and vasculogenesis [15–17]. *BDNF* gene expression has been shown in research to vary by sex, possibly due to sexual dimorphism in hormonal status, enzymatic activities, and body weight [18]. This SNP rs6265 has the potential to influence neuronal adaptation, synaptic plasticity, and motor learning processes, which generate power and coordinate muscle activation during physical activities such as jumps and sprints [19]. Previous studies have shown that *BDNF* rs6265 (A allele) is associated with neuropsychiatric disorders including depression [20], schizophrenia [21], and increased risk of addiction [5], with an increased risk of cardiovascular disease and obesity [22, 23]. Moreover, rs6265 has been implicated in influencing psychological stress response and motivation to exercise [24]. Furthermore, there is evidence suggesting that *BDNF* rs6265 (G allele) is associated with physical activity, motor coordination, memory, cognitive function, visuo-spatial ability [3, 19, 24].

Research on the relationship between *BDNF* genetic variants and the physical capacity of professional athletes has started in recent years [16, 19, 24–27]. However, there is still a lack of consensus regarding the influence of the rs6265 SNP on athletic ability [16, 19, 24–27]. The molecular studies on rs6265 SNP have been conducted with a small number of professional athletes (such as football players, swimmers, and judo athletes), and only in a few populations (Japan, England, Italy, and Turkey). Research involving a small sample size may not fully represent the diversity across different sports, limiting the statistical power to detect a meaningful association between the polymorphism and physical performance phenotype. To address these limitations, we aimed to include larger and more diverse samples of athletes across multiple cohorts and a broader range of sports to determine the association of *BDNF* rs6265 polymorphism with athletic performance. Based on previous findings, we hypothesized that the G allele of *BDNF* is over-represented in elite athletes compared to non-athletic controls. The aim of this research was to investigate the association of the *BDNF* rs6265 (G/A) polymorphism with elite athletic status in four different populations (Japanese and European Caucasian cohorts from Italy, Poland, and Lithuania).

## MATERIALS AND METHODS

### Participants

The study group comprised 1,644 highly skilled athletes and 1,948 controls from four different nations: Japan (JAP), Lithuania (LT), Poland (POL), and Italy (IT). Athletes were divided into three groups according to the duration, nature, and specificity of their sports. The first group consisted of endurance training athletes competing in long distance and duration events (demanding mainly aerobic energy production) and included sports disciplines such as cycling, skiing, swimming, academic rowing, and long-distance track and field athletics (LT: n = 59; POL: n = 96; IT: n = 22; JAP: n = 484). The second group consisted of sprint and power training athletes, whose events require predominantly anaerobic energy sources, and included weightlifting, short distance track and field athletics (LT: n = 87; POL: n = 89; IT: n = 105; JAP: n = 384). The third group consisted of team sports, such as tennis, handball, and football, which requires both anaerobic and aerobic energy production (LT: n = 84; POL: n = 184; IT: n = 50; JAP: n = 0). Notably, the JAP did not have a group of team sports. The inclusion criteria stipulated that the athletes participated in national/international championships (ranked in the top 10 nationally in their respective sports) and must have no history of positive tests in anti-doping controls. The control group included healthy, unrelated individuals with no competitive sports experience (LT: n = 654; POL: n = 371; IT: n = 230; JAP: n = 384).

All procedures were approved by the local Ethics Committees: the Lithuanian Bioethics Committee (protocol code 69-99-111), Poland Ethics Committee at The District Medical Chamber in Gdansk (KB-8/19), Juntendo University (GSHSS 2023-154), and Ethics Committees of the Azienda Ospedaliera Universitaria of Cagliari University (Cagliari, Italy). Written informed consent was obtained from each participant. The study complied with the guidelines set out in the Declaration of Helsinki and ethical standards in sport and exercise science research.

### Genotyping

#### Lithuanian study

For all Lithuanian samples, genomic DNA was extracted from peripheral blood leukocytes using a standard phenol-chloroform extraction method. The concentration and purity of the extracted DNA were measured by a NanoDropR ND-1000 spectrophotometer (NanoDrop Technologies Inc., Wilmington, DE, USA). Genotyping of Lithuanian athletes (n = 230) for the *BDNF* rs6265 genetic variant was performed using an allelic discrimination assay on a 7900HT Fast Real-Time PCR System instrument (Applied Biosystems, Life Technologies, 2012, Carlsbad, CA, USA) with TaqMan SNP genotyping assays (Assay ID: BDNF rs6265 C_11592758_10, Thermo Fisher Scientific UAB, Lithuania) and TaqMan Genotyping Master Mix buffer (Thermo Fisher Scientific UAB, Lithuania). Genotypes were assigned using SDS software v2.3, Applied Biosystems. Genotypes of the Lithuanian non-athlete controls (n = 654) were obtained from Urnikyte et al., 2019, and 2022, which were obtained with the Illumina HumanOmniExpress-12v1.1, the Infinium OmniExpress-24 arrays (Illumina, San Diego, CA, USA), and whole genome sequencing [28, 29].

### Polish study

Genomic DNA was extracted from buccal cells collected with FLO-QSwabs Flocked Swabs (Copan, Italy) using a High Pure PCR Template Preparation Kit (Roche, Switzerland) according to the manufacturer’s instructions. Genotyping of *BDNF* rs6265 SNP was performed using TaqMan SNP genotyping assays (Assay ID: BDNF rs6265 C__11592758_10 (Life Technologies, USA) on a CFX Connect Real-Time Detection System (Bio-Rad, USA). TaqPath ProAmp Master Mix (Applied Biosystems, USA) was used for genotyping, according to the manufacturer’s protocol. CFX Maestro 2.0 Software (Bio-Rad, USA) was used for the visualization and analysis of the amplified products.

### Italian study

Genomic DNA was extracted from a buccal swab using a QIAamp DNA Mini Kit (Qiagen, Hilden, Germany). *BDNF* rs6265 genotyping was performed using the tetra primer amplified refractory mutation system (ARMS) PCR method. This is a high-throughput method, which provides rapid and sensitive *BDNF* rs6265 genotyping. PCR amplifications were carried out in a 25 μl reaction volume containing 50 ng of genomic DNA template, 12.5 μl of PCR master mix (2 ×) and all four primers – P1 (forward): 5’-CCT ACA GTT CCA CCA GGT GAG AAG AGT G-3’; P2 (reverse): 5’-TCA TGG ACA TGT TTG CAG CAT CTA GGT A-3’; P3 (G allele-specific): 5’-CTG GTC CTC ATC CAA CAG CTC TTC TAT AAC-3’ and P4 (A allele specific): 5’-ATC ATT GGC TGA CAC TTT CGA ACC CA-3’. The first set of primers (P1 and P2) amplify the 401 bp region containing the SNP of interest, where- as the second set (P3 and P4) of primers are allele-specific and account for the G > A substitution. The reliability of the tetra primer ARMS-PCR method has been confirmed in previous studies, which demonstrated full concordance with other established genotyping methods such as PCR-RFLP and sequencing, ensuring accuracy and reproducibility in detecting the *BDNF* rs6265 polymorphism [30]. The PCR amplification was carried out in a T100 Thermal Cycler with an initial denaturation temperature of 94°C for 5 min, followed by 30 cycles of 94°C for 45 s, 62.5°C for 60 s, and 72°C for 60 s, and a final extension step of 5 min at 72°C. Stained with Eurosafe (Euroclone S.p.A, Milan, Italy) and visualized in 3% agarose gel, the PCR products indicated successful amplification of all three genotypes (i.e., G/G, G/A, A/A), with G and A allele-specific bands at 253 and 201 bp, respectively.

### Japanese study

Total DNA was isolated from the saliva of each participant using the Oragene DNA collection kit (DNA Genotek, ON, Canada), according to the manufacturer’s instructions. *BDNF* rs6265 genetic polymorphism was genotyped by the use of a real-time thermocycler (Quant-Studio 5, Applied Biosystems, Waltham, MA, USA) with TaqMan SNP Genotyping Assay. Genotypes were called based on TaqMan assay results using QuantStudio Design and Analysis Software (v1.2, Thermo Fisher Scientific).

### Statistical analyses

Statistical data analysis was performed using R Studio 4.2.0 software (main libraries: “HardyWeinberg”, “Car”). Genotype and allele frequencies were calculated. Hardy–Weinberg equilibrium (HWE), chisquare (χ^2^), and Fisher’s exact tests (with a small number of observations) were used to evaluate the differences in allelic or genotype frequencies between athlete and control groups. The odds ratio (OR) with 95% confidence interval (CI) was calculated using binary logistic regression in cases where there was a significant difference in the genotype distribution between groups. The level of significance was set at p < 0.05.

## RESULTS

We performed a collaborative case-control association study (including samples from Lithuania, Poland, Italy, and Japan) and compared the *BDNF* rs6265 genotype and allele distribution across three sports groups (sprint/power, endurance, and team sport) and non-athletic controls. The analysis included three main stages: (I) examining rs6265 genotype/allele frequencies among the three athlete groups (between- and within-group differences in athletes and control groups in each population), and the whole cohort of athletes and control group within each population; (II) examining rs6265 genotype/allele differences of athletes and controls between the European population and Japan (endurance athlete group of three European populations with a Japanese endurance group, etc.); and (III) analysing rs6265 genotype/allele differences of athletes and controls between each population (the Lithuanian endurance group compared to the Polish endurance group, etc.). The first stage data of the genotype and allele distribution analysis are presented in Table 1. The results show that the genotype distributions of controls in all four populations (LT, POL, IT, JAP) were in agreement with the HWE (p > 0.05) (Table 1).

**TABLE 1:** Distribution of genotype and allele frequency of *BDNF* rs6265 polymorphism in the athletes, and controls across four different population groups.

Groups	Allele frequencies (%)			Allele p-value vs controls	Genotype frequencies (%)			Genotype p-value vs controls	HWE p-value
	
	n	G	A		GG	GA	AA		
**Italy**									
Endurance	22	81.8	18.2	0.442	68.2	27.3	4.6	0.640	0.696
Sprint/Power	59	81.4	18.6	0.143	69.5	23.8	6.7	0.079	0.029
Team sports	84	84.0	16.0	0.103	72.0	24.0	4.0	**0.000** *	0.449
*All athletes*	230	82.2	17.8	0.054	70.1	24.3	5.6	**0.041** *	0.024
*Control*	230	75.0	25.0	–	55.9	38.2	5.9	–	0.843

**Poland**									
Endurance	96	87.5	12.5	0.253	77.1	20.8	2.1	0.560	0.641
Sprint/Power	89	87.6	12.4	0.251	77.5	20.2	2.3	0.560	0.531
Team sports	184	86.7	13.3	0.183	74.5	24.5	1.1	0.268	0.420
*All athletes*	369	87.1	12.9	0.084	75.9	22.5	1.6	0.156	0.958
*Control*	371	83.8	16.2	–	71.2	25.3	3.5	–	0.207

**Lithuania**									
Endurance	59	85.6	14.4	0.733	71.2	28.8	0.0	0.457	0.196
Sprint/Power	87	81.6	18.4	0.058	63.2	36.8	0.0	**0.013** *	0.036
Team sports	84	83.3	16.7	0.107	66.7	33.3	0.0	0.064	0.067
*All athletes*	230	83.3	16.7	0.045	66.5	33.5	0.0	**0.001** *	0.002
*Control*	654	87.2	12.8	–	76.0	22.3	1.7	–	0.941

**Japan**									
Endurance	484	58.4	41.6	0.999	34.9	46.9	18.2	0.968	0.442
Sprint/Power	384	58.2	41.8	1.000	33.6	49.2	17.2	0.872	0.820
*All athletes*	868	58.3	41.7	1.000	34.3	47.9	17.7	0.992	0.673
*Control*	821	58.3	41.7	–	34.5	47.6	17.9	–	0.554

* Significant differences between the athlete groups and control group (non-athletes).

Our data showed no statistically significant differences in rs6265 genotype or allele frequency between athlete groups and the control in JAP and POL populations (p > 0.05). However, the results revealed statistically significant differences in genotype frequency distribution between the total group of athletes and the control group in the Italian population (GG/GA/AA: 70.1/24.3/5.6 vs 59.9/38.2/5.9%, p = 0.041), especially in team sport athletes compared with controls (GG/GA/AA: 72.0/24.0/4.0 vs 59.9/38.2/5.9%, p < 0.001) (Table 1). Binary logistic regression analysis showed that the odds ratio (OR) of total IT athletes harbouring the GG genotypes (vs GA+AA, codominant effect) was 1.85 (95%CI: 1.11–3.06, p = 0.017), and OR of the IT team players was 2.03 (95%CI: 0.98–4.21, p = 0.057) compared with controls. Additionally, statistically significant differences in rs6265 genotype frequency distribution were found between the LT total group of athletes and LT controls (GG/GA/AA: 66.5/33.5/0.0 vs 76.0/22.3/1.7%, p = 0.001). The LT athletes were more likely to have the heterozygous GA genotype (vs GG+AA) than the controls (OR 1.75, 95% CI: 1.26–2.44, p = 0.0009). Notably, the absence of the AA homozygous genotype in all LT athletes groups suggests that a rare AA genotype might not be advantageous for athletic performance in Lithuania. Moreover, it was also observed that the GA genotype was more prevalent in LT sprint/power athletes (GG/GA/AA: 63.2/36.0/0.0 vs controls 76.0/22.3/1.7%, p = 0.013), who were 2 times more likely to harbour the GA genotype compared with controls (OR = 2.02; 95%CI: 1.26–3.25, p = 0.003).

Furthermore, we found that the rs6265 genotype and allele frequencies are population-specific and differ among population groups. Genotypes and allele frequencies of the group representing the general population of all three European countries (LT, POL, IT athletes, and control groups when analysed together and separately) were significantly different from the Japanese group (p < 0.001) (Table 2). The rs6265 alternative (minor) A allele is significantly more common in the Japanese population than in Europeans.

**TABLE 2:** Genotype and allele frequencies of the *BDNF* rs6265 polymorphism in athletes and controls from European countries (combined data) and Japan.

Group	*BDNF* (rs6265)	Genotype frequencies				Three European countries vs JAP

		LT+POL+IT		JAP		

		n	%	n	%	p-value
**Endurance**	GG	131	74.0	169	34.92	< 0.001
	GA	43	24.3	227	46.90	
	AA	3	1.7	88	18.18	
	*MAF*: A	49	13.8	403	41.6	

**Sprint/Power**	GG	197	70.1	129	33.59	< 0.001
	GA	75	26.7	189	49.22	
	AA	9	3.2	66	17.19	
	*MAF*: A	93	16.5	321	41.8	

***All athletes***	GG	328	71.6	298	34.33	< 0.001
	GA	118	25.8	416	47.93	
	AA	12	2.6	154	17.74	
	*MAF*: A	186	15.7	724	41.7	

***Control***	GG	818	72.6	283	34.47	< 0.001
	GA	279	24.8	391	47.62	
	AA	30	2.7	147	17.90	
	*MAF: A*	339	15.0	685	41.7	

*MAF*: minor allele frequency; LT: Lithuania, POL: Poland, IT: Italy, JAP: Japan.

After comparing the rs6265 SNP in samples from three European populations (with each other), it was established that the distribution of allele frequencies of non-athlete controls differed significantly between LT and IT (G/A: 87.2/12.8 vs 75.0/25.0%, p < 0.0001), and between POL and IT (G/A: 83.8/16.2 vs 75.0/25.0%, p = 0.005). There was a significantly higher frequency of the G allele in POL and LT than in IT. Notably, the allele frequencies showed no significant differences between total groups of athletes from LT and IT (G/A: 83.3/16.7 vs 82.2/17.8%, p = 0.762) or between LT and POL athletes (G/A: 83.3/16.7 vs 87.1/12.9%, p = 0.076). However, significant differences in allele frequency between POL and IT athletes (G/A: 87.1/12.9 vs 82.2/17.8%, p = 0.038) were observed (Table 3).

**TABLE 3:** P-value of rs6265 allele and genotype differences between the analysed groups.

Groups	Differences in *BDNF* rs6265 (G/A) allele and genotype frequencies P-value											

	LT vs POL		LT vs IT		POL vs IT		LT vs JAP		POL vs JAP		IT vs JAP	

	GF p-value	AF p-value	GF p-value	AF p-value	GF p-value	AF p-value	GF p-value	AF p-value	GF p-value	AF p-value	GF p-value	AF p-value
Endurance	0.692	0.758	0.497	0.729	0.443	0.454	<0.001*	<0.001	<0.001*	<0.001*	0.007*	<0.001*
Sprint/Power	0.015*	0.155	0.011*	1.000	0.259	0.125	<0.001	<0.001*	<0.001*	<0.001*	<0.001*	<0.001*
Team sports	0.244	0.372	0.113	1.000	0.356	0.599	-	-	-	-	-	-
*All athletes*	0.062	0.076	<0.001*	0.762	0.029*	0.038*	<0.001	<0.001*	<0.001*	<0.001*	<0.001*	<0.001*
*Control*	0.081	0.054	<0.001*	<0.001*	0.011*	0.005*	<0.001	<0.001*	<0.001*	<0.001*	<0.001*	<0.001*

* Significant differences between the analysed groups; AF: Allele Frequencies; GF: Genotype Frequencies; LT: Lithuania, POL: Poland, IT: Italy, JAP: Japan.

Examining the distribution of rs6265 SNP genotype frequencies among the three European populations, we found no statistically significant differences in genotype distribution of LT and POL (nor between total athlete and control groups). However, rs6265 GG genotype frequency was significantly higher in POL and LT non-athlete controls than in the IT controls (GG genotype: POL 83.8%, LT 87.5 and IT 55.9%, p < 0.05). Significant differences were found in genotype frequency between total groups of POL and IT athletes (GG/GA/AA: 75.9/22.5/1.6 vs 70.1/24.3/5.6%, p = 0.029) and between LT and IT athletes (GG/GA/AA: 66.5/33.5/0.0 vs 70.1/24.3/5.6%, p < 0.001). In total groups of POL athletes (especially the sprint/power group) the GG genotype is more common compared to LT and IT. However, the GA heterozygous genotype was more frequent in the LT total athlete group (33.5%) compared to the POL (22.5%) and IT athlete group (24.3%) (Table 3).

Despite the described differences, all analysed European populations possess typical European distribution of the studied rs6265 SNP. A high level of the G allele (about 80%) is characteristic of the European population.

Since we found that the Lithuanian and Polish populations were similar according to the *BDNF* rs6265 variant genotyping results, we combined the data and repeated the case-control association analysis. The results showed that the rs6265 SNP genotype distribution of the control group was in agreement with the HWE (p > 0.05), except for the total athlete group (p = 0.035). We determined that the genotype frequencies of professional athletes were statistically significantly different from the control group (GG/GA/AA: 72.3/26.7/1.0 vs 74.7/23.0/2.4%, p < 0.05) (Table 4). The rs6265 GA heterozygous genotype was more prevalent, while the homozygous minor A-allele was less common in elite athletes compared with controls (AA genotype, OR = 0.42; 95%CI: 0.17–1.03, p = 0.05).

**TABLE 4:** Genotype and allele frequencies of the *BDNF* rs6265 polymorphism in joined groups of Lithuanian and Polish athletes and controls.

*BDNF*	Allele frequencies (%)			Allele p-value	Genotype frequencies (%)			Genotype p-value	HWE p-value
	
rs6265 (G/A)	N	G	A		GG	GA	AA		
Endurance	183	84.7	15.3	0.509	70.5	28.4	1.1	0.195	0.193
Sprint/Power	148	86.8	13.2	0.827	75.0	23.6	1.4	0.889	0.683
Team sports	268	85.6	14.4	0.807	72.0	27.2	0.8	0.111	0.080
*All athletes*	599	85.6	14.4	0.719	72.3	26.7	1.0	**0.043** *	0.035
*Control*	1019	86.2	13.8	–	74.7	23.0	2.4	-	0.238

* Significant differences between the athlete groups and control group (non-athletes).

## DISCUSSION

We investigated the association between *BDNF* rs6265 polymorphism and elite athletic status in a large group of elite athletes, comprising Japanese and three cohorts of European Caucasians (Lithuania, Poland, and Italy). This study aimed to perform a collaborative case-control association study and compare the rs6265 genotype/allele distribution across three sports groups (sprint/power, endurance, and team sports) with a non-athletic control group. The main findings of the present research were as follows: (i) the rs6265 SNP was population-specific and differed among population groups (when considering the analysis among all representative study groups). The minor A allele is significantly more common in the Japanese population compared to Europeans, and the G allele (GG genotype) frequency is significantly higher in Poland and Lithuania compared to Italy and Japan; (ii) European elite athletes (especially from LT and POL) are less likely to have the rare AA genotype compared to sedentary controls. Notably, no Lithuanian athletes had the AA genotype; (iii) the GG genotype is more prevalent in IT elite athletes, particularly those in team sports, who are about twice as likely to have the GG genotype compared to controls; (iv) LT athletes are more likely to have the heterozygous GA genotype compared to controls. This is especially true for LT sprint/power athletes, who have double the probability of having the GA genotype compared to controls.

Our results indicate significant genetic differences (according to *BDNF* rs6265) related to athletic performance across four populations and elite sport types, highlighting the potential influence of the *BDNF* rs6265 polymorphism on elite athletic status. However, the findings need to be interpreted in the context of population differences.

According to *gnomAD* v4.1.0 [31], the alternative A allele frequency of the rs6265 SNP varies globally. In Africans/African Americans, the G allele predominates (G/A: 96.7/3.3%), in admixed Americans, there is a high frequency of the G allele (G/A: 85/15%), in Europe the majority has the G allele (G/A: 81.2/18.8%), while in East Asia the frequencies of both alleles are similar due to the predominant heterozygous genotype (G/A: 55.4/44.6%). In this study, the A allele of the rs6265 SNP was also significantly more prevalent in the Japanese population. In Lithuania, Poland, and Italy, the genetic variation of the rs6265 SNP aligns with typical European patterns. Lithuania and Poland show no significant differences in rs6265 SNP, likely due to their partial genetic similarity stemming from geographical and historical connections [32]. The differences observed between the European populations under study and the Japanese population suggest ancestry-relevant heterogeneity. This indicates that population-specific associated SNP is more likely to have been subject to natural selection, potentially leading to different genetic influences on phenotypic variation. The distinct allele frequencies and genotype distributions among these populations high-light the complex interplay between genetic and environmental factors, shaping the traits associated with elite athletic performance. BDNF in muscle is synthesized by myofibers, and also by satellite cells and blood vessel endothelial cells. The muscle-derived BDNF regulates the metabolism of myofibers and muscle regeneration (autocrine/paracrine activities), and serves as a hormone to communicate with other tissues [13, 33]. BDNF is known to regulate neuronal survival, growth, and neurogenesis, and the *BDNF* rs6265 SNP is related to serum BDNF concentration in response to exercise [3, 17]. This SNP may likely affect neuronal adaptation and thus the ability to activate the appropriate muscles and generate more power during physical activities [19]. Moreover, the rs6265 SNP influences the psychological stress response and motivation to exercise. This has a clear impact on the positive or negative thinking of the individual during the competition. Thus, the athlete can be more effectively guided to better manage their emotions and stress to achieve optimal results.

There is evidence that individuals with the GG genotype of rs6265 may confer an advantage in sprint and power sports, while the A allele might not be favourable for sports [19]. Previous studies showed that the rs6265 AA genotype is associated with an increased risk of cardiovascular disease, obesity, and neuropsychiatric disorders (such as depression, schizophrenia, and vulnerability to psychological stress) [20–23]. Asai et al. examined Japanese elite male swimmers and judo athletes and found that the frequency of A allele carriers was lower in judo athletes, suggesting that judo athletes had stronger stress resistance. In this study swimmers had a higher frequency of heterozygous GA genotype, suggesting that they are superior in motor control and motor learning [24]. As the G allele is associated with a greater abundance of exercise-induced serum BDNF concentration, research findings suggest that GG genotyped individuals with potentially enhanced neuromuscular characteristics perform better in tasks that require anaerobic abilities. Recent research has highlighted the significance of the *BDNF* rs6265 variant in team sport athletes with both great aerobic and anaerobic capacity. Team sports players (e.g. footballers) perform intermittent, repetitive, high-intensity actions, and those requiring explosive power. These activities depend on both anaerobic and aerobic energy production pathways. Murtagh et al. investigated 535 elite male youth football players and 151 controls at different stages of maturity and found that the rs6265 GG genotype was associated with enhanced anaerobic capacity, including horizontal power, acceleration, and sprint performance [19]. A study involving sub-elite Australian football players revealed that individuals with the rs6265 heterozygous GA genotype exhibited enhanced performance in skill tests related to kicking and handballing [27]. The results of our research partially confirm these associations in Italian and Lithuanian populations. The findings indicated that the rs6265 GG genotype is more prevalent in Italian professional team athletes, who are more likely to harbour this genotype compared with controls. In the Lithuanian population, a heterozygous GA genotype advantage in the sprint/power group includes athletes requiring anaerobic capacity. However, research on Turkish volleyball players and Caucasian elite male rugby athletes did not find significant associations between physical performance characteristics and the *BDNF* rs6265 variant among these ethnically diverse athletes [34].

There is evidence suggesting that BDNF may contribute to skeletal muscle fibre-type topology, and is important for the survival and maturation of motor neurons and fast-twitch muscle fibres [35]. Furthermore, BDNF contributes to the homeostasis of energy metabolism. After binding to the kinase B receptor associated with tropomyosin (TrkB), BDNF causes a molecular signal that leads to increased fat oxidation, decreases the size of adipose tissue, and increases insulin sensitivity [36]. Moreover, BDNF acts as a cardioprotective agent, helping protect the heart muscle from damage. This protection is crucial for athletes whose heart muscles endure significant stress. BDNF protein expression correlates with oxidative stress and with VEGF (vascular endothelial growth factor A) expression, which contributes to the regulation of angiogenesis [16]. BDNF and VEGF play critical roles in maintaining the health and function of heart muscles in athletes, helping them cope with high workloads, recover effectively, and ensure long-term heart health and wellness. Therefore, in the context of sports and physical activities, BDNF plays a crucial role in regulating cardiovascular functions and immunomodulatory processes, and maintaining energy balance [33, 37–40]. This is achieved by affecting cellular responses to glucose and insulin, mitochondrial activities, differentiation of thermogenic tissues, and cognition enhanced by exercise [36]. The *BDNF* rs6265 polymorphism impacts gene expression and protein stability, potentially explaining the observed correlations between performance characteristics and an athlete’s profile.

It is important to note that athletic performance is a complex trait influenced by numerous genetic and environmental factors. The *BDNF* genetic variants are just one piece of this intricate puzzle. We believe that the results of the present research are overall reasonable, as all of the following criteria of the case-control association study were met: 1) clear presentation of phenotypes: cases were well defined as elite athletes); 2) ethnic matching: participants (both athletes and non-athlete controls) within each cohort were ethnically matched, covering populations from Poland, Lithuania, Italy, and Japan; 3) accurate genetic investigation, which was conducted correctly, accurately, and without bias; 4) the genotype frequencies in the control group of the four cohorts were consistent with HWE. While HWE was not met in some athlete groups, it is emphasized that for genetic association studies, it is essential to meet HWE only in the control group, as these individuals are supposed to represent the general population.

However, we acknowledge some limitations of the study. First, we did not collect direct phenotype measures of athletes’ sprint, power, or endurance (e.g., muscle mass, maximal oxygen consumption, motor coordination, reaction time, speed). However, our inclusion criteria stipulated that all athletes had competed at the national or international level, which would not be possible without the physiological characteristics associated with elite athletic phenotypes. Second, we studied a single SNP, and we recognize that genetic association studies represent only the first steps toward understanding the genetic factors influencing physical performance traits. However, candidate gene association studies are crucial for guiding new analyses, such as those incorporating modern DNA technologies and bioinformatics. These studies help to further analyse the heritability of physical capacity and work toward the application of genomic research in practice.

## CONCLUSIONS

In this study, the differences regarding the *BDNF* rs6265 variant observed between the European populations and the Japanese population suggest ancestry-relevant heterogeneity. *BDNF* rs6265 is more likely to have been subject to natural selection, potentially leading to different genetic influences on phenotypic variation including elite athletic performance. Our results highlight the potential influence of the *BDNF* rs6265 polymorphism on elite athletic status in Italian (GG genotype) and Lithuanian (GA genotype) populations. Looking to the future, more studies are needed to determine *BDNF* rs6265 effects on gene expression and function, and its interaction with environmental factors across different populations throughout the world.

## Data Availability

All relevant data are within the manuscript and its supporting information files.
